# Nanometer Scale Hard/Soft Bilayer Magnetic Antidots

**DOI:** 10.1186/s11671-016-1302-3

**Published:** 2016-02-13

**Authors:** Fanny Béron, Andreas Kaidatzis, Murilo F. Velo, Luis C. C. Arzuza, Ester M. Palmero, Rafael P. del Real, Dimitrios Niarchos, Kleber R. Pirota, José Miguel García-Martín

**Affiliations:** Instituto de Física Gleb Wataghin (IFGW), Universidade Estadual de Campinas (UNICAMP), Campinas, SP 13083-859 Brazil; Institute of Nanoscience and Nanotechnology, NCSR “Demokritos”, Aghia Paraskevi, Attiki, Athens, 15310 Greece; Instituto de Ciencia de Materiales de Madrid (ICMM), Consejo Superior de Investigaciones Científicas (CSIC), Madrid, 28049 Spain; IMM-Instituto de Microelectrónica de Madrid (CNM-CSIC), 28760 Tres Cantos, Madrid Spain

**Keywords:** Exchange-spring magnets, Structured magnetic thin films, Magnetic antidot arrays, First-order reversal curve (FORC), Magneto-optical Kerr effect (MOKE), 75.60.-d, 75.70.Ak, 78.20.Ls

## Abstract

The effect of arrays of nanometer scale pores on the magnetic properties of thin films has been analyzed. Particularly, we investigated the influence of the out-of-plane magnetization component created by the nanopores on the in-plane magnetic behavior of patterned hard/soft magnetic thin films in antidot morphology. Its influence on the coupling in Co/Py bilayers of few tens of nanometer thick is compared for disordered and ordered antidots of 35-nm diameter. The combination of magneto-optical Kerr effect (MOKE) and first-order reversal curve (FORC) technique allows probing the effects of the induced perpendicular magnetization component on the bilayer magnetic behavior, while magnetic force microscopy (MFM) is used to image it. We found that ordered antidots yield a stronger out-of-plane component than disordered ones, influencing in a similar manner the hard layer global in-plane magnetic behavior if with a thin or without soft layer. However, its influence changes with a thicker soft layer, which may be an indication of a weaker coupling.

## Background

Magnetic thin films are used nowadays in a wide variety of applications, such as cores in small current transformers, media for magnetic storage of information, and transducers in magnetic sensors, to name a few. In particular, exchange-coupled hard/soft magnetic bilayers are magnetic stacks combining a hard magnetic layer with high coercive field and a softer one with lower coercivity. They can be used as model systems to study the properties of exchange-spring magnets [1]. In 2005, a particular type of exchange-coupled hard/soft bilayers began to be developed, where the layers have perpendicular anisotropies [2, 3]. This appears to be a promising candidate system both for magnetic recording, because it reduces the required writing field and allows tuning the system anisotropy [4, 5], and for spin transfer torque RAM (STT-RAM) and spin-torque oscillators (STO) devices, through the possibility of tuning the magnetization tilt angle [6].

On the other hand, the inclusion of artificial defects in the bilayers helps to engineer their magnetic properties. One common example of such morphology modification is antidot arrays, where a regular pattern of nanoholes acts as local pinning sites for the magnetization [7–9]. As a result, the magnetic anisotropy, the coercivity, and the remanence can be tailored by modifying the antidot diameter, the separation, and order among them, as well as the film thickness [10–13]. These systems can be used in several applications, such as magnonic crystals [14], magnetoplasmonics [15], and bio-sensors [16].

It has been found that the stray field reduction, due to the nanopore presence in the magnetic thin films, induces a magnetization deflection not only in-plane but also out-of-plane (OOP) [17]. Moreover, the applied magnetic field in-plane direction can considerably modify the size of the magnetic domains exhibiting one particular perpendicular magnetization component. For a perfectly hexagonal ordered Fe antidot array, this size is either of the interpore distance order (hundreds of nm) or covering an area of many nanopores (several μm), depending if the in-plane magnetizing field is applied along the next-nearest neighbor or nearest neighbor directions, respectively [18]. Therefore, modifying the antidot pattern could lead to distinct consequences of an OOP magnetization component on the system magnetic behavior.

In this work, our main objective is to study the influence of the OOP magnetization component on hard/soft bilayer magnetic antidots made of materials without magnetocrystalline perpendicular anisotropy. For this purpose, we fabricated bilayers of 20-nm-thick Co with up to 27-nm-thick permalloy (Py) thin films on nanoporous alumina templates with pore diameter of around 35 nm [19]. Two kinds of templates were used: completely disordered and short-range ordered ones, the latter exhibiting pores locally ordered in a hexagonal pattern. For comparison, single layer antidots and continuous thin films of each studied system (i.e., single layers and bilayers) were also prepared.

Magneto-optical Kerr effect (MOKE) was chosen as magnetization detection technique due to its high sensitivity, since the small film thicknesses lead to a low magnetic signal. It allows a rapid measurement of the surface magnetization, therefore eliminating the need to remove the large diamagnetic contribution arising from the substrate. It has already been used to characterize both exchange-spring systems [20–24] and antidot arrays [18, 25, 26].

On the other hand, major hysteresis curves only yield the global magnetization behavior. To investigate complex systems containing several magnetization reversal mechanisms, first-order reversal curve (FORC) technique is a powerful method that has already been successfully applied to several nanostructured systems (i.e., array of nanodots [27, 28], nanopillars [29], and nanowires [30–32], among others). When the result analysis is based on the classical Preisach model [33], it yields the statistical distribution of the elementary irreversible magnetization reversal processes, called hysterons [34]. Even for magnetic systems that do not meet the required conditions for the classical Preisach model, such as magnetic antidots, the FORC distribution can give highly valuable information [35–37]. Moreover, we have already investigated the specific influence of an OOP magnetization component (created by nanopillars) on a Py antidot array by means of FORC technique [38]. Finally, this characterization technique also proved its utility to investigate exchange-spring magnets, both for multiphase systems [39–43] and multilayer thin films [44, 45].

Even if the FORC technique use has considerably spread out during the last 5 years, it is still largely limited to measurements performed on quasi-static magnetization detection, such as vibrating sample magnetometer (VSM). Few studies took advantage of the MOKE superficial magnetization measurement combined with FORC technique [46], mainly due to the special cares one needs to consider when implementing FORCs acquisition on a magnetic field-swept system. A method was recently proposed in this sense [47]. However, we decided to base our experimental FORC acquisition on the principles followed during the FORC implementation on an AC induction magnetometer [48].

In this work, we report on the effect of the magnetic antidot pore order on the coupling between hard (Co) and soft (Py) thin films with few tens of nanometer thickness. A surface characterization is first presented, where the pore diameter and order are measured by means of atomic force microscopy. It is followed by a magnetic characterization, performed by magnetic force microscopy (MFM) and by coupling MOKE and FORC techniques. It is shown that the antidot morphology induces an out-of-plane magnetization component that depends on the pore order and that the soft layer thickness may affect the hard/soft magnetic layer coupling.

## Methods

### Sample Fabrication

#### Substrate Preparation

The continuous thin films were deposited on Si <100> substrates with a 500-nm-thick thermal silicon oxide. Ordered and disordered antidot arrays were fabricated using nanoporous alumina templates [49]. A DC voltage of 40 V was applied on previously electropolished high-purity aluminum disks immersed in oxalic acid solution at 3 °C. Disordered antidot arrays were produced using alumina templates anodized once, while a two-step anodization process (with a first anodization of 24 h) was performed for the short-range ordered ones [50]. Planarization of the alumina templates was carried out through ion milling with Ar^+^ ions during 16 min using 500 V acceleration potential and 200 μA/cm^2^ current density.

#### Thin Film Deposition

All thin films were deposited using an ultra-high vacuum DC magnetron sputtering system. The sources were tilted at 25° with respect to the substrate normal axis, while the substrate was rotating in order to obtain a homogeneous deposition on the surface of the templates and minimize the amount of material entering into the pores. We first placed a 2-nm-thick Cr thin film as buffer layer. Then, the magnetic films were deposited: either single layers (20-nm-thick Co or 27-nm-thick Py (Ni_80_Fe_20_) thin films), either bilayers starting with 20-nm-thick Co thin film as the hard layer, followed by Py as the soft layer (9 or 27-nm-thick). The obtained systems were denoted as Co*t*_Co_Py*t*_Py_, where *t*_Co_ and *t*_Py_ represent the nominal thickness of Co and Py thin films, respectively. Finally, a 5-nm-thick Pt thin film completed the thin films deposition, as a capping layer preventing oxidation. For determining the deposition rate of all the materials used, test samples have been prepared and their thickness was measured by means of X-ray reflectivity measurements.

### Characterization

#### Atomic Force Microscopy

The morphology of the initial templates and the antidot arrays was characterized by atomic force microscopy (AFM) in non-contact mode using a Bruker Dimension Icon microscope with super sharp probes (about 3 nm radius) from Next-Tip (http://www.next-tip.com/). We compared the pore diameter and surface profile height variation before and after surface planarization by ion milling and after thin films deposition. We calculated the two-dimensional self-correlation and fast-Fourier transform (FFT) of the AFM images of the initial templates, in order to characterize the pore order and interdistance.

#### Micromagnetic Simulations

Micromagnetic simulations of the samples were performed using the freely available OOMMF software (http://math.nist.gov/oommf/) . Actual 3 × 3 μm^2^ AFM images were treated and used as masks to recreate disordered and short-range ordered antidots morphologies, although only the central 1 × 1 μm^2^ region will be shown to avoid the magnetic features that are mainly due to the edges. The cell size was kept as 4 × 4 × 5 nm^3^. Co and Py exchange constants *A* were taken as 30 × 10^−12^ and 13 × 10^−12^ J/m, respectively, while an intermediate value (20 × 10^−12^ J/m) was used for the Co/Py interface. Saturation magnetization values were 1400 kA/m (Co) and 860 kA/m (Py), while magnetocrystalline anisotropy was considered as negligible for both materials, due to their polycrystalline structure. The remanent state was calculated after fully saturating the system along the plane.

#### Magnetic Force Microscopy

The magnetic microstructure of the antidot arrays was imaged by magnetic force microscopy (MFM). A Bruker Dimension Icon microscope was used with commercial MFM probes (Bruker MESP). The phase-imaging double-pass tapping-mode was used for obtaining MFM images: after recording the surface topography during the first pass, the tip was lifted of 60 nm and the magnetic contrast was recorded. As the MFM tip was magnetized along the axis vertical to the sample, the observed contrast originates from magnetic charges on the sample surface. In all cases, we imaged the demagnetized state of the antidot arrays.

#### Magneto-optical Kerr Effect

Thin film magnetization *M* was acquired by means of longitudinal MOKE, on a NanoMOKE2™ setup. The magnetic field *H* was applied along the sample plane, while the laser beam (nominal laser spot diameter of 3 μm, wavelength of 658 nm, and power of 7.5 mW) makes an angle of 45° with it. Each magnetization curve was independently corrected for coil remanence and Faraday rotation. Both major hysteresis curves and first-order reversal curves (FORCs) (see next section for details) were measured. The hysteresis curves were acquired using a sinusoidal magnetic field (27 Hz, maximum field higher than 100 Oe) and averaging the obtained signal between 500 to 1000 times, with at least 1200 points per curve.

#### MOKE-FORC

FORCs are minor curves beginning at different reversal fields (*H*_*r*_) and ending when the sample is saturated. Due to the particularities of FORCs, the MOKE acquisition parameters were modified. In order to keep constant both the field sweep rate and the working frequency, we used a triangular field signal with an amplitude covering from the reversal to the saturation fields, but that remains saturated during the required time at the saturation field. Since the beginning of the FORC is right after the triangular field minimum, the working frequency was kept low (1.013 Hz), to avoid a field signal distortion near the reversal field. For each FORC measurement, between 350 and 500 curves were acquired, averaged between 20 and 30 times. Before the FORC distribution *ρ*_FORC_ calculation [34], the obtained curve was processed: the data for the non-increasing field (due to the low working frequency) were removed, before the field step interval was adjusted to be half of the reversal field step [51].

## Results and Discussion

### Surface Characterization

**Fig. 1:**
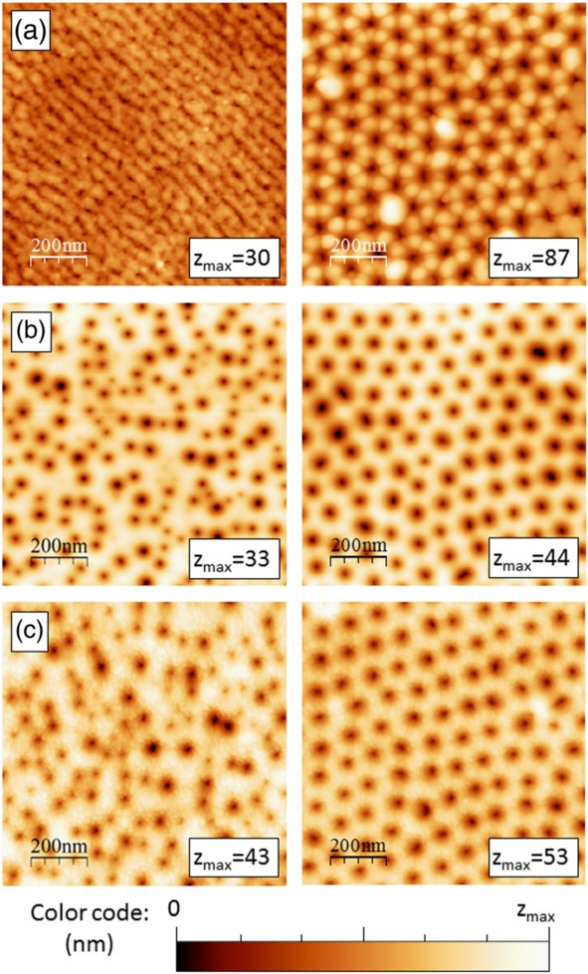
Typical AFM images of the antidot arrays surface. **a** Initial templates before planarization. **b** Templates after planarization. **c** Final samples after thin film deposition (Co20Py27). *Left*: disordered antidots, *right*: ordered antidots. The maximum height variation in the AFM image is indicated (nm)

The as-obtained short-range-ordered and disordered alumina templates are quite different (Fig. 1a). In the disordered case, the pores appear smaller, irregular, and not well delimited, while they are clearly visible in the short-range ordered templates. The maximum height variation (*z*_max_) is more than the double in the latter case: 87 nm in comparison to 30 nm. This difference can arise from two identifiable sources. First, the disordered antidot surface is the as-initiated first anodization result. Therefore, the pores are beginning to form, while they will rearrange themselves in a hexagonal pattern of uniform diameter at the alumina/aluminum interface, which is below the observed surface. The small pore opening at the surface prevents the AFM tip to completely enter inside the pore, yielding a smaller height variation. In addition, the six-fold crests around each ordered pore, as typically observed for two-step anodized alumina templates [36], substantially increase the measured *z*_max_ value.

**Fig. 2:**
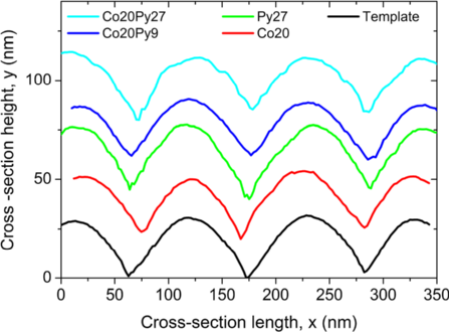
Typical height cross-sections of the ordered antidot arrays, before and after thin film deposition, as extracted from AFM images. The profiles have been artificially displaced along the vertical direction for the sake of clarity

After performing the surface planarization, the discrepancy in the maximum height observed between both templates almost disappears (Fig. 1b). Since the ion milling etches the surface, it opens the disordered pores, while removing the ordered pore crests. It results in similar height variations and pore diameters, of the order of 40 and 35 nm, respectively. In all cases, the surface profile is not greatly affected by the thin film deposition (Fig. 1c). To adequately compare the effects of the thin films, height cross-sections were extracted from AFM images, before and after deposition (Fig. 2). The height variations remain in the same range but slightly reduce with the total film thickness. The measured profile is always limited by the pyramidal shape of the AFM tip, but no significant differences are observed. Therefore, we can conclude that we successfully fabricated antidot films that follow the substrate morphology.

**Fig. 3:**
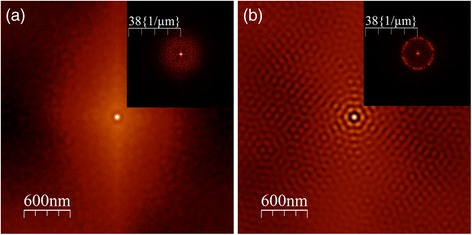
Typical two-dimensional self-correlation spectrum for **a** disordered and **b** ordered templates, calculated from the AFM images before thin films deposition. *Inset*: respective spectrum two-dimensional FFT

The alumina template pore order can be characterized by the two-dimensional self-correlation spectrum of the AFM images (where a bright peak denotes for a similar pattern repetition) as well as by their corresponding FFT images (Fig. 3). Both spectra exhibit an intense signal in its center, produced by the presence of pores of comparable size. For the disordered template, the rest of the self-correlation spectrum is diffused and the FFT only exhibits a halo, corroborating the lack of pore order. On the other side, a clear short-range hexagonal pattern is visible on the spectrum of the ordered template, and the FFT image exhibits six maxima. In this case, an average distance between first neighbors can be extracted and its value is 103 nm. Additionally, even if the disordered spectrum FFT does not present a sharp signal, the width of the halo suggests that the maximum first neighbors distance is similar in both antidot geometries. Since the pore diameters are also in the same range, the main difference between antidot substrates is their pore order, passing from completely disordered to a short-range hexagonal order.

### Magnetic Characterization

**Fig. 4:**
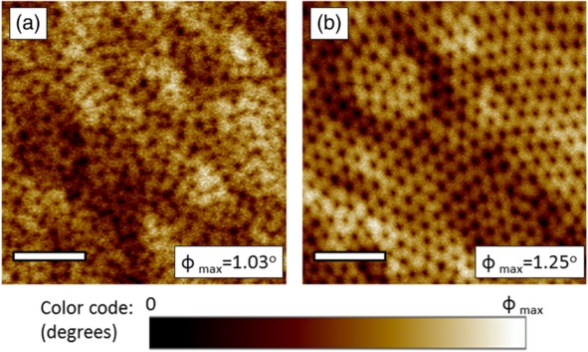
MFM imaging (phase detection) of the Co20Py27 sample magnetic structure in in-plane demagnetized state. **a** Disordered array. **b** Ordered array. The *scale bar* is 500-nm long

First of all, in order to verify the existence of an OOP magnetization component while the antidot arrays are magnetized in-plane, MFM imaging of their magnetic structure has been performed (Fig. 4). As the MFM tip is magnetized along the axis perpendicular to the sample surface, the measurement is sensitive to the OOP magnetization component of the antidot array magnetic structure, which is evidenced by the bright and dark contrast appearing in both images. This contrast is more intense in the case of the ordered antidots, indicating a stronger OOP magnetization component (Fig. 4b).

**Fig. 5:**
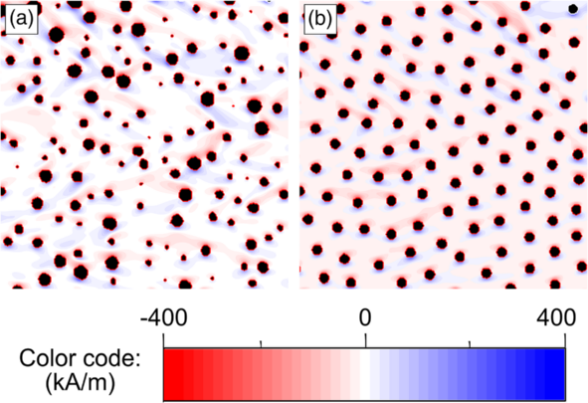
Top view of the 1 × 1 μm^2^ central region of the micromagnetic simulation result of Co20Py30 (magnetization along z direction, pores represented in *black*). **a** Disordered antidots. **b** Ordered antidots

The presence of such an intense OOP component may appear to be counter-intuitive, as shape anisotropy in thin films results in in-plane magnetization. However, taking into account the total magnetic film thickness of the sample (Co20Py27: 47 nm) and the diameter (35 nm) and periodicity (103 nm) of the antidots, it is deduced that the magnetic elements between two neighboring antidots have an aspect ratio lower than 2:1. Thus, magnetic charges at the antidot edge and at the film surface are of comparable importance [18], explaining the development of an OOP magnetization component. This is shown in Fig. 5, where micromagnetic simulations for Co20Py30 in the form of a disordered antidot array and an ordered one are depicted, with the color code associated to the z-component (perpendicular to the plane) of the spins. Such component is almost negligible for a continuous film (not shown), whereas it can be clearly seen for the antidot arrays, especially for the ordered one. It is worth noticing that we are not mimicking the domains observed in Fig. 4, since domain formation is the result of the energy minimization of the whole samples: we are only simulating how the existence of nanoholes helps to develop an OOP component.

**Fig. 6:**
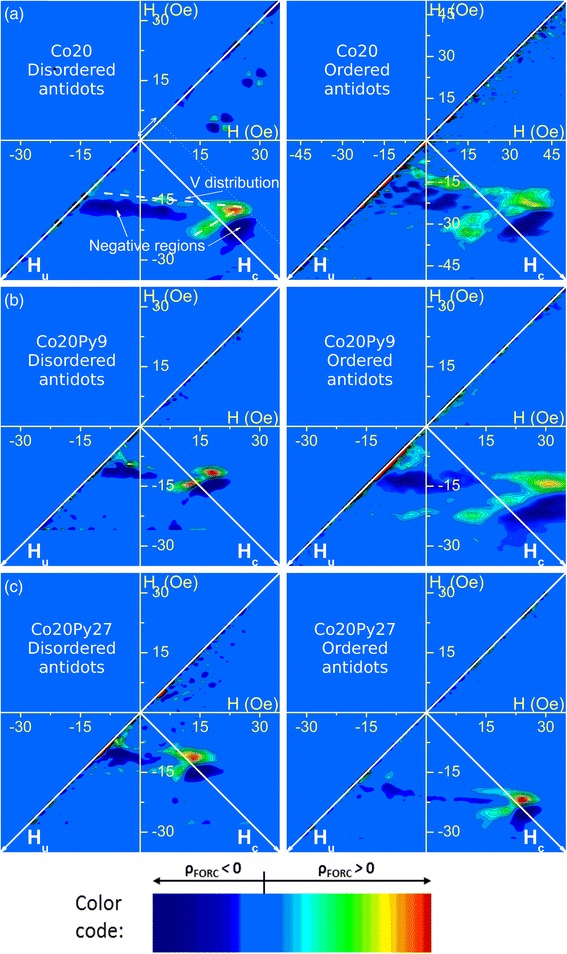
Longitudinal MOKE-FORC diagrams of the antidot arrays. **a** Co20. **b** Co20Py9. **c** Co20Py27. *Left*: disordered antidots, *right*: ordered antidots

In this work, we want to investigate the influence of this OOP magnetization component on the in-plane behavior of nanostructured hard/soft bilayers. In this sense, FORC method applied along the plane is highly advantageous: in addition to be easy to measure, it informs about both the presence and the effects of an OOP component [38]. While all MOKE-FORC results for continuous films exhibit only one distribution (not shown), suggesting a strong hard/soft coupling, antidot results present at least two positive FORC distributions, in addition to negative regions (Fig. 6). The general FORC distribution shape remains similar in all these cases: a sharp V distribution created by the link of two or more peaks, opened toward left, with negative regions between the V legs and below the lower leg, and finally, the V junction is dislocated above the *H*_*c*_ = (*H* − *H*_*r*_)/2 axis (enhanced on Fig. 6a, left side). It differs from some previously observed FORC results of antidots, which mostly appear as one slightly curved distribution localized below the *H*_*c*_ axis [36]. In this case, this FORC distribution shape has been proved to arise from a collection of hysterons characterized by a coercivity distribution and submitted to a small magnetizing interaction field [52]. However, since these antidot arrays had not been planarized before deposition, the interaction field had been attributed to the remaining crests around the nanopores [36]. In fact, our results are similar not only to what observed for comparable preparation process [37] but also to what exhibited by antidot arrays combined with nanopillars [38]. In addition, the observed particular FORC distribution cannot be related to the presence/absence of magnetic coupling between the hard and soft layers, since the Co20 antidots present a similar pattern.

**Fig. 7:**
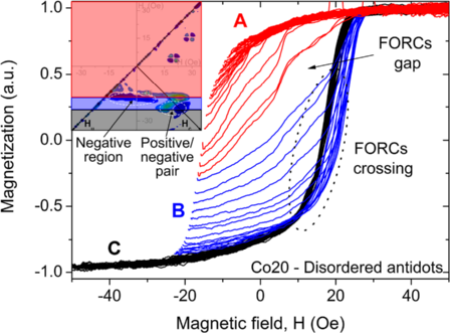
Representative longitudinal MOKE first-order reversal curves of an antidot array, evidencing the three behaviors observed (Co20 on disordered template). *Inset*: FORC diagram indicating the respective FORC regions created by the different FORCs groups (A: *red*, B: *blue*, C: *black*)

Since the FORC distributions contain clear negative regions, they cannot be interpreted as a statistical distribution of hysterons. Two characteristic features can be distinguished on them: a positive/negative pair distribution, creating the lower V part, and the negative region between the V-shape (encircled on Fig. 7 inset). Both arise from sharp magnetic behavior transitions, which can be evidenced on the FORCs. All antidot sets of FORCs exhibit three different behaviors: the first FORCs group (A) remains inside the major curve path, while the second one (B) exits it, thus creating a crossing with the FORCs coming from an almost saturated state (C) (Fig. 7). The transition between A and B FORC groups leads to a gap in the FORCs set. Since the magnetization derivative function of *H*_*r*_ (∂*M*/∂*H*_*r*_) significantly decreases in this region, it results in a negative FORC region along *H*_*r*_ = 16 Oe. The second transition, between B and C FORCs groups, is different. FORC crossover yields two opposite distributions: a positive and a negative one before and after the FORC intercept, respectively, due to ∂*M*/∂*H*_*r*_ changing sign. This feature has also been experimentally observed in the in-plane behavior of antidots with nanopillars inducing an OOP magnetization component [38]. We assumed that both transitions exhibited by the antidots under study are consequences of the presence of an OOP magnetization component. Since the OOP orientation changes during magnetization reversal accordingly to its magnetic history [18], its switch first creates the FORC gap, due to the effective field modification. The FORC crossover, for its part, may be explained by the vanishing of the OOP magnetization component at the *H*_*r*_ field value between B and C groups. Assuming that its direction remains the same after positive and negative saturation, it will be identical in A and C FORC groups and opposite in B one. Therefore, the FORC behavior transitions indicate a modification of the OOP magnetization direction in the system.

**Fig. 8:**
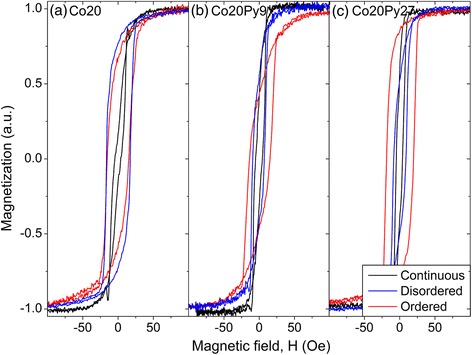
Longitudinal MOKE major hysteresis curves for **a** Co20 **b** Co20Py9 **c** Co20Py27

**Fig. 9:**
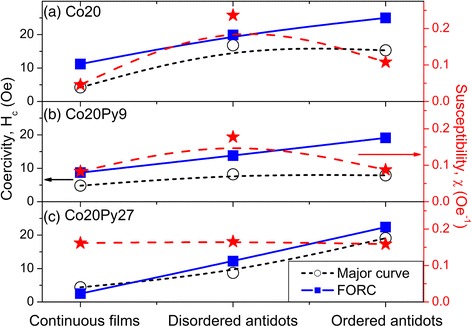
Evolution of the coercivity (*left axis*) and susceptibility (*right axis*) values with the pore order. **a** Co20. **b** Co20Py9. **c** Co20Py27

Micromagnetic simulations showed that the addition of an OOP magnetization component, through the addition of small nanopillars, influences the antidot array global in-plane behavior [38]. When it is aligned along the same direction in a given region, both the susceptibility and the global coercivity decrease due to the resulting OOP dipolar field. This field also favors a reversible magnetization reversal, which further lower the major hysteresis curve coercivity (global one), in comparison with the one extracted from the FORC diagrams, taken as its position on the *H*_*c*_ axis. In order to experimentally correlate the OOP magnetization component intensity with the antidot array pore order, we measured the in-plane major hysteresis curves (Fig. 8), from which we extracted the susceptibility and the global coercivity (Fig. 9). For both the Co20 single layer and the Co20Py9 bilayer, in addition to the expected coercivity increase when passing from a continuous thin film to an antidot array, both the susceptibility and global coercivity are lower for the ordered template, compared to the disordered one. Moreover, the difference between the coercivity extracted from FORC diagrams and the global one from the major curves is larger for the ordered antidots than for the disordered ones (Fig. 9a, b). These results agree with a stronger OOP magnetization component in the ordered antidot morphology, as deduced from MFM characterization.

On the other hand, for a thicker soft layer in the bilayer, the trends differ: for Co20Py27, the susceptibility is almost constant, while the global coercivity, higher for ordered than disordered antidots, remains near the one extracted from FORC distributions (Fig. 9c). Even if the similarity between antidots FORC distribution patterns suggests that all the systems contain an OOP magnetization component, a similar tendency is observed when comparing the disordered/ordered FORC distributions: both Co20 and Co20Py9 exhibit a more spread distribution with additional positive peaks, while it is only shifted toward higher coercivity for Co20Py27, keeping the same pattern. Therefore, it seems that a good hard/soft magnetic layer coupling was achieved for small Py thickness (9 nm), whereas a larger soft layer thickness (27 nm) may have weaken the coupling. This fact is evidenced through MOKE results, since due to the exponential decay of the light in metals, the signal probed comes mainly from the now uncoupled Py layer.

## Conclusions

In summary, hard/soft bilayer magnetic antidots have been successfully fabricated on nanoporous alumina templates. It has been shown that the antidot morphology induces an out-of-plane magnetization component in these disordered and short-range ordered systems. Such OOP component is stronger for ordered arrays than for disordered ones. The influence of this phenomenon on the in-plane global behavior of the hard/soft bilayer antidots depends on the soft layer thickness.
